# Stainless Steel Voltammetric Sensor to Monitor Variations in Oxygen and Humidity Availability in Reinforcement Concrete Structures

**DOI:** 10.3390/s21082851

**Published:** 2021-04-18

**Authors:** Ana Martínez-Ibernón, Josep Lliso-Ferrando, José M. Gandía-Romero, Juan Soto

**Affiliations:** 1 Interuniversity Research Institute for Molecular Recognition and Technological Development (IDM), Universitat Politècnica de València, 46022 Valencia, Spain; jollife2@alumni.upv.es (J.L.-F.); joganro@csa.upv.es (J.M.G.-R.); juansoto@qim.upv.es (J.S.); 2 Department of Architectural Construction, Universitat Politècnica de València, 46022 Valencia, Spain; 3 Department of Chemestry, Universitat Politècnica de València, 46022 Valencia, Spain

**Keywords:** voltammetric sensors, durability, concrete, corrosion, monitoring

## Abstract

The present work presents the results obtained with a stainless steel (SS) voltammetric sensor to detect variations in humidity (H_2_O) and oxygen (O_2_) availability in concretes. First, studies in solution were run by preparing several solutions to represent the different conditions that can be found in concrete pores. Second, the sensor’s response was studied by varying O_2_ availability by argon or synthetic air bubbling. Then concrete conditions with different degrees of carbonation were simulated using solutions with a pH between 13 and 8.45. After characterization in solution, a study by means of concrete samples with several water/cement ratios (0.6, 0.5 and 0.4) was performed, in which sensors were embedded and studied under different O_2_ and H_2_O saturation conditions. The obtained results revealed that with the voltagram, it is possible to evaluate O_2_ availability variation from the slopes of the lines identified logarithmically in the voltagram for the obtained cathodic sweeping. All the results obtained with the sensor were correlated/validated by standard assays to characterize porosity in hardened concretes.

## 1. Introduction

The follow up and forecasting of reinforced concrete structures’ (RCS) service lives are some of the main recent objectives for reducing economic and environmental costs that are a direct result of inefficient maintenance actions, repairs that could have been minimized, and even demolishing buildings which could have been avoided if suitable preventive measures had been taken. Moreover, control and efficacious estimations would have helped to ensure people’s safety.

In order to develop models that allow optimum follow up and forecast structures’ service lives, it is necessary to know which phenomena threaten their durability. With RCS, one of the main problems is rebar corrosion [1,2,3,4]. The basic most extensive service life model related to this phenomenon was developed by Tutti [1,5]. This basic model distinguishes two periods: the first is the period of initiation, which includes the time from when aggressive agents penetrate the concrete matrix to the time when they reach rebars and bring about their depassivation; the second is the period of propagation, which covers the time from when rebars depassivate to when corrosion starts developing. The structure’s service life ends when an unacceptable degree of corrosion is reached.

These durability models applied to a given structure can be built by taking measurements of the structure in real time with monitoring systems. One of the most promising systems is that formed by embedded sensors, which is why recent studies have focused on developing them [3,4,6,7,8,9,10,11,12,13].

When following up corrosion processes in structures, sensors can be made that center on the period of initiation by detecting the presence of agents that trigger rebar depassivation by, for example, concrete carbonation or the presence of chlorides [2,4,14]; on the other hand, sensors can be used that assess the period of propagation either by quantifying the substances directly involved in corrosion processes like O_2_ and H_2_O, or stating the rebar corrosion status [15]. Developing voltammetric multi-sensor systems that assess both the aforementioned periods is another possibility and would, therefore, allow complete service life models to be developed.

The voltammetric sensors operation is based on the application of potential signals continuously modified as a linear function of time. Each potential generates an electric current response related to a specific reaction, which involves the interest agents (oxygen, water, chlorides, etc.). By means of the current analysis as a function of the potential, we can quantify these agents [16]. This is clearly an advantage over other type of sensors, such as potentiometric or resistance sensors, because it allows for developing more accurate models in order to quantify different analytes contained in the electrolyte.

Apart from seeking sensor systems’ effectiveness, it is fundamental to create autonomous and very reliable low-cost and high-durability systems. This is why it is so important that the material used to make sensors meets these requirements.

Stainless steel (SS) use is widespread in industry thanks to its stability in many aggressive environments, and also its low cost compared to other metals [17]. This alloy presents excellent mechanical and corrosion resistance and is a good conductor of both heat and electricity [18]. Thus, its use has grown in recent years in different sectors, such as energy, pharmaceuticals, shipping, and construction [18,19,20].

In short, we understand SS to be an iron carbon alloy that contains a minimum of 10.5% chrome. Although this steel is called stainless, it does not mean that it does not corrode, rather a protective layer of chrome oxide forms on its surface instead of common rust. If under normal atmospheric conditions this chrome oxide layer deteriorates, it is immediately formed again by chrome combining with oxygen (O_2_) in environmental atmospheres to protect the metal from poorly stable and highly soluble iron oxide formation [21].

Today this metal has been employed to manufacture sensors used in the food industry [22] and those embedded in concrete [8,23]. With concrete, Correia, et al. [6] demonstrate the effectiveness of using an SS electrode to detect O_2_ variations inside concrete. In the same article, Correia, et al. [6] also mention how the degree of concrete saturation is observed by Rs measurement (resistance to the ions circulating through concrete [23]) by the impedance spectroscopy technique. The article by McCarter and Vennesland [8] refers to SS being used as a reference electrode and counter electrode in sensor systems to control RCS corrosion. The article by Xu, et al. [24], presents a new kind of stainless steel (SS) galvanic sensor system for the study of rebar corrosion in different pore solution conditions. Tabatabai and Aljuboori [25] developed a resistance sensor made with SS in order to detect ice and water on roads and bridges.

On the other hand, many authors propose that O_2_ reduction in SS is associated with the zones in which Fe(II) compounds act as absorption centers, promoting the oxygen reduction reaction [6,21,26,27,28].

Bearing this in mind, developing a voltammetric sensor would be interesting to monitor RCS’ service life with this metal because the effectiveness of sensors to detect variations in O_2_ availability inside the RC matrix has been proven. Therefore, being able to develop O_2_ availability quantification models by sensor measurements would be very interesting indeed. O_2_ is one of the main agents that induces rebar corrosion in RCS [4], [6,29,30]. Furthermore, unexpected increase in O_2_ availability can mean a physical defect of the concrete cover in RCS, such as the formation of cracks. It could identify indirect reactions as a result of the concrete carbonation, because this phenomenon produces the pH change of the concrete pore solution. The change of pH significantly affects the oxygen reduction in the SS surface [21].

Moreover, this metal’s low cost and its good services would allow sensor systems to be produced that cover large surfaces and offer excellent durability at a much lower cost than if noble metals were employed.

Our objective was, therefore, to assess the possibilities of using SS voltammetric sensors embedded in concrete to develop a sensor system with SS. To do so, we assessed their reactivity to the different conditions that could come into play in concrete (carbonation, chloride penetration, variations in O_2_ availability, etc.) by employing solutions that simulate concrete pore solution. Then we assessed the behavior of sensors when they were embedded in concrete by varying O_2_ availability conditions and humidity before checking if cracks in concrete could be detected, and even different cracking patterns, by these sensors [31].

The objective of the analysis herein performed was to understand how the SS sensor works to know its limitations, to define simple correlations, and to identify its potential to discriminate changes in O_2_ availability and humidity by bearing in mind that they are most important in the corrosion reaction of RCS.

## 2. Materials and Methods

In order to fulfil the proposed objectives, we defined the following study phases:

- Phase (1) Study in solution. In this preliminary phase, the processes that took place on the SS electrode surface when immersed in solution were studied, which represent the different conditions that could come into play in concrete. The obtained results were compared with those found in the literature.
- Phase (2) Studies performed with the SS sensor embedded in conventional concrete. SS voltammetric sensors were embedded in conventional concrete samples at different water/cement ratios (w/c). Samples were studied under distinct O_2_ availability conditions.

The sections below explain in detail the experiments that were run in each phase and how SS sensors were produced.

### 2.1. Studies in Solution

Two different O_2_ availability conditions in solution with electrodes immersed in 0.1 m KOH solution (this solution simulates the concrete pore solution’s pH under normal conditions, pH ≈ 12.5) were studied:

- In the first situation, O_2_ availability in solution was reduced by argon bubbling for 30 min before testing
- In the second situation, KOH solution was O_2_-saturated by bubbling synthetic air for 30 min before testing. Synthetic air was used to prevent an atmospheric CO_2_ solution and to avoid the employed solution’s carbonation.

After this study, different solutions were prepared to simulate the different conditions that could come into play in the concrete pore solution (tests run with these solutions are always done in synthetic air):

- Solutions 0.1 m KOH (pH = 13) and 0.1 m NaOH (pH = 12.7) to simulate non-carbonated concrete [32]
- 0.1 m Na_2_CO_3_ solution (pH = 11.45) to simulate the initial degree of carbonation [32]
- 0.1 m NaHCO_3_ solution (pH = 8.35) to simulate carbonated concrete [32]
- Solution 0.1 m NaOH +Na_2_CO_3_ Ci m (pH = 12.7), where Ci came in these concentrations: 0.02 m, 0.05 m and 0.1 m. The purpose was to evaluate how the presence of carbonates influenced the obtained results.
- Solution 0.1 m NaOH (pH = 12.7) by adding NaCl at 0.5 m
- Solution 0.1 m NaOH (pH = 13) to simulate non-carbonated concrete by adding H_2_O_2_ at 0.02 m, and to assess the possible appearance or stabilization of this intermediate product in reducing O_2_, as suggested in the literature

The results were compared to those found in the literature to understand how the O_2_ reduction reaction takes place on the SS electrode and how these conditions can affect it, as we focused on this reaction in the present study.

#### The Applied Electro-Analytical Techniques

The following electro-analytical techniques of cyclic voltammetry and impedance spectroscopy were applied by Autolab PGSTAT10 equipment and the Nova 1.11 software.

The test configuration applied with both these techniques was the 3-electrode configuration. The same SS 304 wire to manufacture sensors was employed as the working electrode (0.8 mm diameter, 12 cm long) was used. An INOX plate was utilized as the auxiliary electrode (105 cm^2^ surface). The reference electrode was a saturated calomel electrode (SCE), while the potential values herein presented referred to the SCE.

The cyclic voltammetry technique was applied to the range within which O_2_ reduction took place and was a range of sufficiently negative potentials to induce the process [23]. Here the intention was only sweeping in the area of the negative potentials. We made sure to not exceed the “critical pitting potential” during anodic sweeping where the passivity of SS steel was lost and became active [26,33].

Potential sweeps commenced from the OCP (open circuit potential or the system’s equilibrium) cathodically until −1.2 V, before returning to 0 V (anodic sweeping) and ending back at the OCP to make the system as least polarizing as possible. The sweep rate was 20 mV/s. The aim of such sweeping was to disturb the real O_2_ availability inside the concrete matrix as little as possible.

The ohm drop in the voltagrams was corrected with the Rs value obtained by the impedance spectroscopy technique with the same 3-electrode configuration. The range of sweeping frequencies went from 100,000 Hz to 1000 Hz. Sweeping at high frequencies was justified by simplifying the system similarly to Randles simple equivalent electrical circuit (Rs-Rp/Cdl). The effective value of the applied tension was 10 mV.

### 2.2. Studies Performed with the Sensor Embedded in Concrete

**Figure 1:**
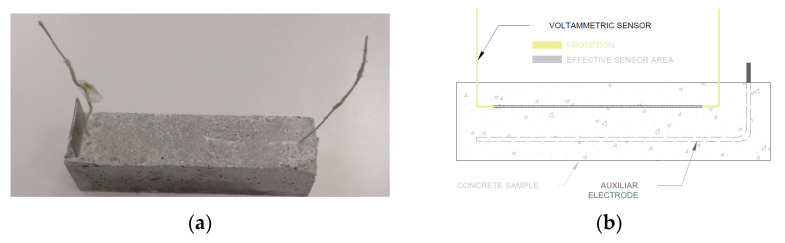
(**a**) Picture of a sample with an embedded SS sensor; (**b**) Outline of the configuration of the SS sensors embedded in a concrete sample.

In order to manufacture SS sensors, a 0.8 mm-diameter SS 304 wire was used. The sensor’s working surface was limited by covering the ends with waterproof polyurethane paint. Sensors’ average unsupported length was 12.3 ± 0.5 cm. They were made U-shaped to easily insert them into samples (Figure 1).

L-shaped 3 cm-wide SS plates were used as counter electrodes. The average surface of these employed counter electrodes was 113.34 ± 2 cm^2^, which was about 37-fold bigger than the surface of SS sensors to ensure that those reactions taking place on the counter electrodes would not interfere with the response obtained in the electro-analytical tests [34].

These sensors were embedded in prismatic concrete samples (4 × 4 × 16 cm^3^) made with three different types of concrete, which are set out in Table 1.

Figure 1 illustrates how sensors were placed inside concrete samples. Sensors were placed at a depth of more or less 1 cm at the top of samples.

To confer the study statistical reliability, three mixes were produced with each concrete type and two samples were made per mix. Table 2 includes the number of samples per dosage.

Samples were left in a curing chamber for 28 days before being dried in an oven at 40 °C for 24 h. Then they were left under environmental conditions to perform the experimental part.

The variables established in the experimental part were:

- Concrete type. Samples made of three different concrete types and, therefore, with distinct porosity, were used, which would imply differences in O_2_ availability in the vicinity of the electrode when samples were under similar environmental conditions
- O_2_ availability. Samples were left under four different environmental O_2_ availability conditions:
  ∘ Atmospheric conditions (ATM), O_2_ molar fraction (x_O2_) 0.21
  ∘ Partial air pressure condition, 500 mbar (500 mbar), O_2_ molar fraction (x_O2_) 0.105
  ∘ Vacuum conditions (VAC), the O_2_ molar fraction (x_O2_) came very close to 0
  ∘ H_2_O saturation conditions (SAT), the available O_2_ in the concrete matrix was limited by the gas solution capacity in the concrete pore solution.

In accordance with the O_2_ availability conditions, the experimental part was divided into two subparts (see Table 3).

#### 2.2.1. Study in the Non-Saturated State

**Figure 2:**
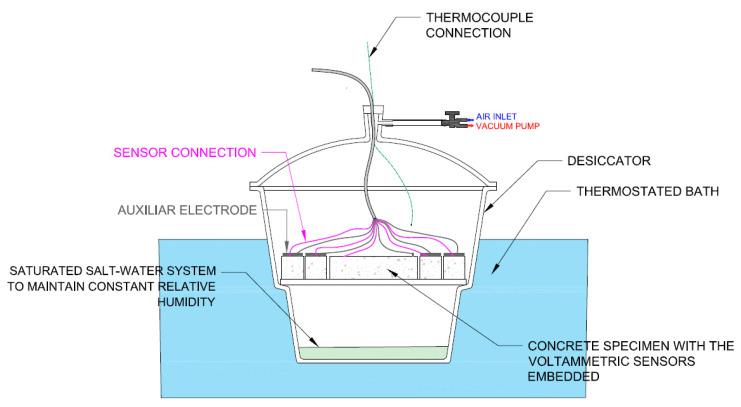
Test setup.

The system described in Figure 2 was set up in the non-saturated state to submit samples to different O_2_ availability conditions. In this state, partial O_2_ pressure was regulated inside the desiccator by a vacuum pump. To keep temperature constant, the desiccator was left in a thermostatic bath and the temperature inside the desiccator was left at 23 °C ± 2 °C. To achieve constant relative humidity (RH) conditions, saturated salt solution was used to keep RH at around 84.5% ± 0.3 (saturated KCl solution) [35]. With an approximately constant temperature and RH during tests, constant environmental absolute humidity conditions were achieved, which enabled us to ensure that the humidity which samples could absorb did not differ due to the environmental conditions, rather owing to their own porosity.

The following test sequence was repeated three times per dosage:

1. Vacuum conditions: To homogenize the initial behavior of the sensors embedded in the different samples, the studied samples were subjected to a vacuum for 12 h. After finishing the adaptation period, the aforementioned electro-chemical studies were carried out.
2. Atmospheric conditions: Once the tests under the vacuum conditions ended, synthetic air was allowed to enter the desiccator. When a 1-bar pressure was achieved inside, 60 min were allowed for the system to stabilize before testing began.
3. Partial 500-mbar air pressure: when the tests under atmospheric conditions ended, the pressure inside the desiccator was lowered until vacuum conditions were achieved. At this point, 30 min were allowed before letting air in until a sTable 500-mbar pressure was accomplished. After a 15-min wait, argon was injected until the 1-bar pressure was recovered. In this way, the O_2_ molar fraction inside the desiccator diminished to half in relation to the working atmospheric conditions. Measurements were taken on sensors after 1 h under 500 mbar conditions.

A preliminary study was performed which demonstrated that the 1-h adaptation time before starting each test series sufficed to ensure the reproducibility of the obtained results.

While testing, the temperature was controlled inside the desiccator by the thermocouple placed inside it.

After repeating the test sequence for 3 days, samples were left in a closed container under 100% humidity conditions to prevent concrete carbonation.

#### 2.2.2. Study in the Saturated State

**Figure 3:**
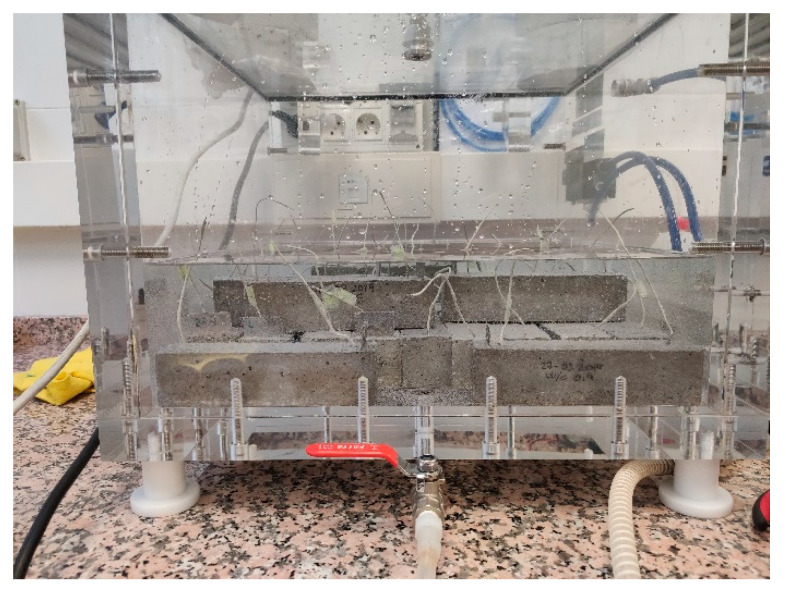
Samples immersed in the vacuum chamber.

In order to achieve the saturated state, all the samples were placed inside a vacuum chamber (Figure 3). Vacuum conditions were set up in this chamber and lasted 1 h, after which time H_2_O was allowed to enter and completely cover samples. The system was left under these conditions without breaking the vacuum for another 1-h period. After this time, the vacuum was broken, and samples were left immersed for 48 h before the test series commenced under these conditions.

Tests were carried out under these conditions on three consecutive days and for all the dosages at the same time.

#### 2.2.3. Applied Electro-Analytical Techniques

The same techniques as in Phase I were applied. The only difference on this occasion was that work was done in a 2-electrode configuration because the setup in the desiccator and the test sequence did not allow for placing a reference electrode inside the system that came into contact with each studied sample’s surface. This configuration was acceptable because the counter electrode had a much bigger surface than the working electrode (WE) [34].

#### 2.2.4. Concrete Characterization Tests

The results of these standardized tests were used to support the results obtained with the sensors.

The following tests were done:

- Hardened concrete tests, Part 3: determining sample strength to compression (UNE 12390-3:2009). Resistance to compression was determined at 28 days (fck). One cylindrical sample (10 cm diameter, 20 cm high) was prepared for each mass per dosage, with nine samples in all. The equipment used was Ibertest MEH-3000.
- Determining H_2_O absorption, density, and H_2_O accessible porosity (UNE 83980:2014). One cylindrical sample (10 cm diameter, 5 cm high) was prepared for each mass per dosage. Nine samples in all.
- Determining H_2_O penetration depth under pressure (UNE 83-309-90). One sample (15 cm diameter, 30 cm high) was produced for this test for each mass per dosage. Nine samples in all.
- Determining air permeability (UNE 83981:2008). One cylindrical sample (15 cm diameter, 5 cm high) was prepared for each mass per dosage. To perform the test, the sides of the samples were covered with sealing paint. The air permeability coefficient was obtained (k). Nine samples in all.
- Determining electrical resistivity (ρ): direct method (reference method) (UNE 83988-1:2008). A prismatic sample (4 × 4 × 16 cm^3^) was prepared for each mass per dosage. Nine samples in all.
- Mercury injection porosity (MIP) test (ASTM-D4404-10). This test allows information to be acquired about the volume of interconnected pores and their size distribution. These tests were performed by the ITC of the Universitat Jaume I (Spain). The MIP method consists of injecting mercury at different pressures. The volume of absorbed mercury at each pressure is recorded so that the volume of absorbed mercury within a certain range of pressures is associated with a given access size range.

The equipment used was Mercury Posorimeter Micromeritics, AutoPore IV (9500). In order to perform tests, a sample was taken from specimen nuclei. Before running tests, samples were dried at 100 °C.

Table 4 shows the mean values obtained during the characterization tests, as well as the coefficient of variation (CV), defined as the quotient between the standard deviation and the average value.

In short, the lower the w/c ratio, the higher the fck for 28 days, and the less the H_2_O accessibility inside the matrix for both absorption and pressure; at a higher strength, concretes present higher electrical resistivity in the saturated state and less air permeability.

## 3. Results

**Figure 4:**
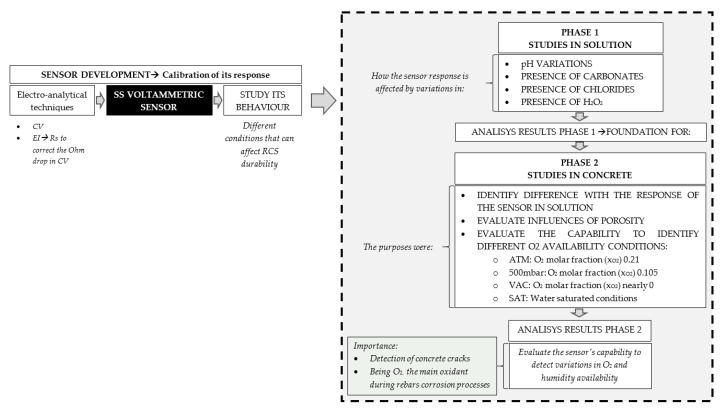
Scheme of the mechanism for the experimental results.

In order to show the study results to clarify the following mechanism, Figure 4 is presented.

### 3.1. Phase 1: Studies Performed in Solution

We observed that the SS sensor in solution behaved slightly differently to that embedded in concrete. This agrees with what Correira et al. [6] reported. This difference might be due to the diffusion phenomena in concrete being very different to that in solution, which could influence the passive oxide layer that forms on the SS surface.

In line with previously published works [23], the nomenclature used in voltammetry graphs of electrical potentials includes ΔE_WE_ for those cases in which the ohm drop is corrected and work is done with a 3-electrode configuration, and ΔV_WE_ for the case when the ohm drop is corrected but work is done with a 2-electrode configuration.

#### 3.1.1. Reducing O_2_ on the SS Electrode’s Surface

**Figure 5:**
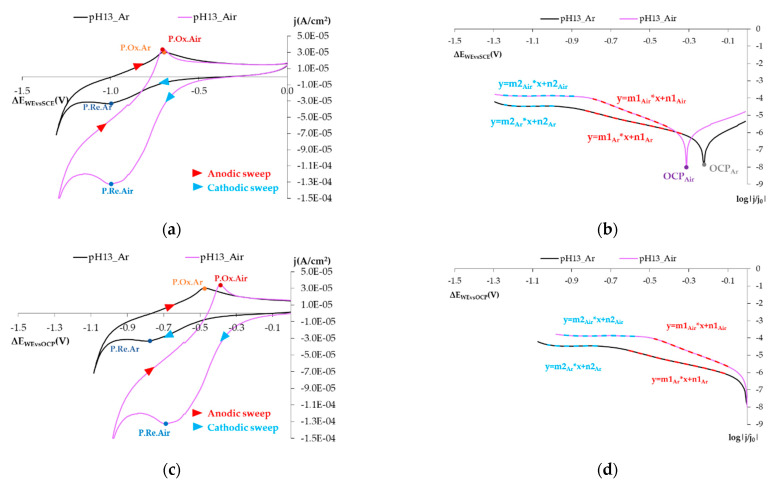
(**a**) Voltagrams obtained with the SS sensor under different O_2_ airing conditions for the 0.1 m KOH solution. Potentials referring to the reference SCE. (**b**) Voltagram of Figure (**a**) expressed logarithmically (log|j/j_0_|), only cathodic sweeping. (**c**) Voltagrams obtained with SS sensor under different O_2_ airing conditions for the 0.1 m KOH solution. Potentials referring to the corresponding OCP. Black line: Tests in an argon atmosphere. Lilac line: Tests in a synthetic air atmosphere. (**d**) Voltagram of Figure (**c**) expressed logarithmically (log|j/j_0_|), only cathodic sweeping. Black line: Tests in an argon atmosphere. Lilac line: Tests in a synthetic air atmosphere.

According to Babic and Metikos-Hukovic [26] and Bösing et al. [36], the peaks observed in Figure 5a,c during anodic sweeping (P.Ox.) are associated with the electroformation of Fe(OH)_2_ layers and their oxidation to form FeOOH layers, while the peaks noted during cathodic sweeping (P.Re.) are associated with a reduction in these products. The Fe(OH)_2_ and FeOOH layers form on a pre-existing protective Cr_2_O_3_ layer [26,36].

Figure 5a,c depicts the voltagrams obtained using the SS sensor when moving from an airless atmosphere (the black line) to an aired atmosphere (the lilac line). Figure 5a shows the potentials vs. SCE and Figure 5c shows the potentials vs. OCP.

These figures show a large difference in current density in the zone where a reduction peak appeared (P.Re.). Bearing in mind both test conditions, this increase could only be due to the reduction in O_2_ (because N_2_ is an inert gas). This agrees with many authors in the literature who propose that O_2_ reduction in SS is associated with the zones in which Fe(II) compounds act as absorption centers [6,21,26,27,28].

The reaction of the reduction in the O_2_ dissolved on the SS surface can take place with four (1) or two (2) electrons [17,18,26,27,37,38].

O_2_ + 2H_2_O + 4e^−^ → 4OH^−^ (1)

O_2_ + 2H_2_O + 2e^−^ → 2OH^−^ + H_2_O_2_ (2)

Which reaction predominates apparently depends on the electrode’s surface composition and also on the conditions it is under [6,37,38].

Figure 5b,d is the logarithmic representation of the cathodic sweeping zone of the experiments indicated in Figure 5a,c. The j/j_0_ logarithmic value is represented on the *Y*-axis, where j_0_ is the reference current density of 1 A/cm^2^ and j is the density obtained at each potential applied in A/cm^2^.

With these representations, the following can be stated:

- The open circuit potential (OCP) shifts to the left if the atmosphere is aired.
- In this representation, two straight-lined sections appear. These lines are associated with O_2_, H_2_O_2_, and H_2_O reduction processes. The following can be deduced from the study:
  ∘ The first straight line is that which varies the most with changes in O_2_ availability, and the value of its slope in the airless atmosphere goes from −2.630 V^−1^ to −4.272 V^−1^ (Table 5). The variation in the intercept is slight. This variation in the representation of potentials versus the SCE is 6.8% and 4.5% versus the OCP (Table 5). These minor variations do not enable us to establish significant differences between both states.
  ∘ The second line is practically horizontal. The value of this line’s slope seems to be independent of the solution’s airing (Table 5). This plateau aspect indicates that it is due to a limitation in the speed of the material’s transport process.

Parameter m (slopes of straight lines) is an interesting parameter because it does not depend on either the reference being used in the technique or the electrode’s surface. This means that it is very efficient as a comparative parameter among several tests run with different sensors (demonstrated in Appendix A).

#### 3.1.2. Influence of pH on the SS Electrode

Poorly soluble carbonates form when concrete is carbonated, mainly by the reaction with calcium hydroxide, which lowers the hydroxide concentration in the concrete pore solution and, therefore, also its pH, with pH values coming close to neutrality (pH ≈ 8) [34].

According to the study performed by Babic and Metikos-Hukovic [26], pH influences the surface conditions of SS, hence the importance of studying this influence because the electrode’s surface conditions impact O_2_ reduction owing to passive oxide layer formation [6,37,38].

**Figure 6:**
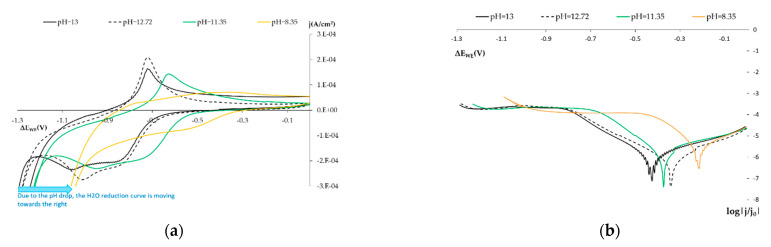
(**a**) Voltagrams obtained with the SS sensor at various pHs. (**b**) Voltagrams expressed logarithmically, only cathodic sweeping. Black line: 0.1 m KOH solution (pH = 13). Dashed black line: 0.1 m NaOH (pH = 12.72). Green line: 0.1 m Na_2_CO_3_ solution (pH = 11.45). Orange line: 0.1 m CNaHO_3_ solution (pH = 8.35).

In order to assess how variations in pH influence the sensor’s response, Figure 6 compares the results obtained with the solutions that simulated the three different pH conditions of the concrete pore solution:

- Normal conditions (pH 13 and pH 12.72).
- The initial carbonation state (pH of 11.45).
- Carbonated concrete (pH 8.35).

Figure 6a shows how the sensor’s response changes with pH. As pH lowers (with pH close to neutrality), the shown peaks in both the anodic and cathodic sweepings shift to the right and their current density (j) lowers. This agrees with the study on SS performed at different pHs by Babic and Metikos-Hukovic [26].

As medium basicity diminishes, the OH^-^ concentration drops, Fe(OH)_2_ formation is inhibited and, therefore, the current density of the associated peak lowers.

Fe^2+^ + 2OH^−^ → Fe(OH)_2_; (3)

Bearing in mind that some authors believe the O_2_ reduction occurs on Fe(II) compound layers, the alteration to these layers that results from varying pH may influence the measurement taken by the sensor to characterize O_2_ availability. Figure 6b and Table 6 show the difference in the four pH values studied by means of the logarithmic representation. In all cases, line 2 is seen as a practically constant horizontal line with a similar intercept value for this line. We can see that the slope values for line 1 are similar. Therefore, we can state in a first instance that varying pH would not significantly affect identifying the availability of O_2_ and humidity.

We also observe how at the start of the curve associated with H_2_O reduction, less negative potentials appear as pH lowers, which is consistent with the H_2_O pourbaix.

#### 3.1.3. Influence on the Electro-Chemical Behavior of Other Chemical Species

In concrete pore solution, we find both hydroxides and carbonates, and occasionally chlorides in aggressive environments. Another interesting reaction from the mechanistic point of view is that H_2_O_2_ as the O_2_ reduction reaction on the SS surface can occur with two or four electrons to generate OH^−^ or H_2_O_2_ [17,18,26,27,37,38]. Depending on the electrolyte conditions and the metal surface, one product or another being produced is favored. With H_2_O_2_, its presence can affect O_2_ reduction on the steel surface [17,27,37,38].

This section aims to identify if the presence of carbonates, chlorides, and H_2_O_2_ influence in the O_2_ reduction on the steel surface , and the solutions were:

- Influence of carbonates: Solution 0.1 m NaOH + Na_2_CO_3_ Ci m (pH = 12.7), with Ci concentrations of 0.02 m, 0.05 m and 0.1 m.
- Influence of chlorides: Solution 0.1 m NaOH (pH = 12.7) by adding NaCl at 0.5 m.
- Influence of H_2_O_2_: Solution 0.1 m NaOH (pH = 12.7) by adding H_2_O_2_ at 0.02 m.

**Figure 7:**
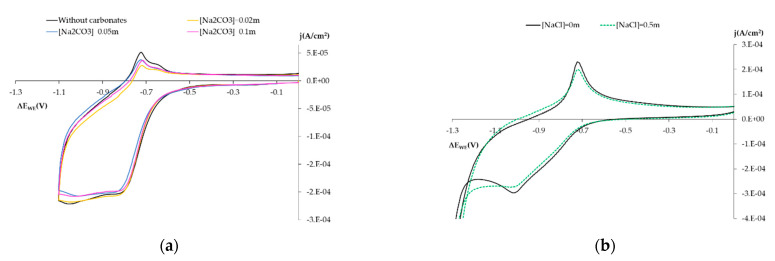
(**a**) Voltagrams obtained with the the SS sensor in solution NaOH 0.1 m + Na_2_CO_3_ Ci m. Black line: Ci = 0. Yellow line: Ci = 0.02 m. Blue line: Ci = 0.05 m. Pink line: Ci = 0.1 m. (**b**) Voltagrams obtained with SS sensor in solution NaOH 0.1 m + NaCl Ci m. Black line: Ci = 0. Dashed green line: Ci = 0.5 m.

From the results in Figure 7, we note how the presence of carbonates and chlorides barely affected the results obtained with the SS electrode, provided that pH is not affected.

According to the study by Correia, et al. [6], the surface conditions of steel embedded in concrete can be affected by the presence of Cl anions.

**Figure 8:**
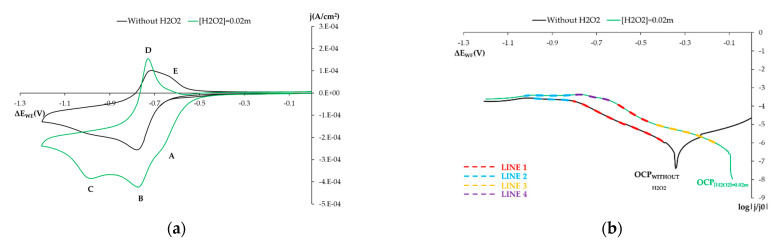
(**a**) Voltagrams obtained with the SS sensor in 0.1 m NaOH solution and 0.02 m H_2_O_2_. (**b**) Voltagrams of the Figure a logarithmically represented, only cathodic sweeping. Black line: without H_2_O_2_. Green line: With [H_2_O_2_] = 0.02 m.

Figure 8a shows the voltagrams obtained with the stainless steel (SS) electrode at pH 12.72 (solution 0.1 m NaOH) to reduce dissolved oxygen (O_2_) before (voltagram in black) and after adding (in green) 0.02 m of H_2_O_2_ to the system. The voltammetry obtained when H_2_O_2_ is lacking, which shows that reduced O_2_ is associated with peak B, appears at this pH at the potential of −0.78 V vs. SCE. This peak is followed by a shoulder of slight current density (peak C) at a maximum potential of −1 V vs. SCE. During anodic sweeping, D peaks appear (−0.74 V vs. SCE), as does a subsequent E shoulder at approximately −0.6 V vs. SCE. Peak D may be related to the oxidation process of the species obtained after reducing molecular O_2_ (peak B).

The addition of H_2_O_2_ to the studied system brought about a morphological variation in the voltagram with a new peak appearing (A), as well as a marked modification in the currents recorded for the other peaks.

It is well known that H_2_O_2_ can act as a very weak acid (pKa = 11.6) to generate the perhydroxyl anion (HO_2_^−^) and to release protons to the medium. Under our working conditions (pH 12.72), the non-dissociated H_2_O_2_ concentration was only 9.5% of the 0.02 m added to the system, while the remaining 90.5% came in the form of HO_2_^−^. It is also known that on surfaces like that of iron, this species’ decomposition is catalyzed in molecular O_2_ and H_2_O.

The voltagram obtained by adding H_2_O_2_ to the system (Figure 8a, green line) seems to fall in line with the above reasoning. We associate shoulder A, which came before the B peak that we related to the reduction in molecular O_2_, with the reduction in non-dissociated H_2_O_2_. Peak B current density significantly increased due to the O_2_ generated by the HO_2_^−^ dismutation catalyzed by the sensor’s surface. Under these conditions, the current density of the third peak (C) also significantly increased. This was associated with the bioelectronic HO_2_^−^ reduction in the presence of one H_2_O molecule, which led to the formation of species OH^−^. When the anodic cycle was run, peak E disappeared in the presence of H_2_O_2_ and peak D intensified, possibly because the processes overlapped, which would mask the slower process associated with peak E.

The way the system behaved in the CV study is reflected in the logarithmic representations of the current’s intensity vs. the potential. The system’s OCP value significantly shifted, new slopes appeared (Figure 8b), and the slope m1’s absolute value increased when H_2_O_2_ was added (Table 7). This higher value indicates the increased presence of O_2_, a finding that falls in line with what we discuss. O_2_ availability increased due to the greater HO_2_^−^ dismutation caused by this species increasing when H_2_O_2_ was added to the solution, as discussed.

### 3.2. Phase 2: Studies Performed with the Sensor Embedded in Concrete

The objective of the analysis carried out in this study phase was to understand how the sensor embedded in concrete works, to know its limitations, to define simple correlations, and to identify the sensor’s discrimination potential.

**Figure 9:**
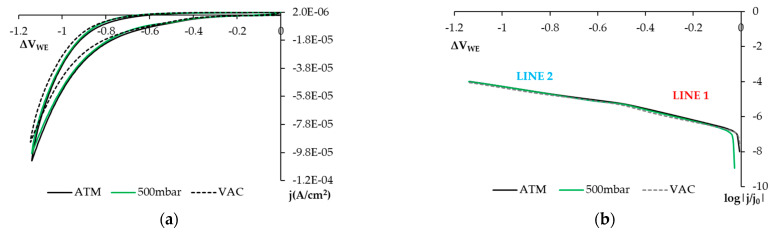
Results of cyclic voltametry with the SS sensor embedded in a hardened concrete sample of w/c = 0.6 under atmospheric conditions (black solid line, ATM), under partial air pressure condition, 500 mbar (green solid line, 500 mbar) and VAC conditions, with practically no O_2_ (dashed line, VAC) using the 2-electrode technique. (**a**) Voltagram in which ohm drop is corrected. (**b**) Voltagram of cathodic sweeping represented logarithmically that corresponds to Figure a.

The article by Correia, et al. [6] mentions how the passive oxide layer that forms in the SS embedded in concrete differs from that produced in solution. Hence the obtained voltagrams differ even under similar pH conditions, as seen in the voltagram shown in Figure 9a (black solid line). In concrete in the non-saturated H_2_O state, we cannot distinguish the peaks associated with the formation of Fe(OH)_2_ and FOOH layers.

The cited article [6] explains how the passive oxide layer that forms on the surface of the sensor embedded in concrete favors O_2_ reduction. The cited authors state that a bigger metal surface covered by Fe(II), and not by solution, probably exists.

In the voltagrams in Figure 9a, obtained in our study, we see how current density diminishes on the cathodic sweeping curve under the 500 mbar and VAC conditions, i.e., when O_2_ availability is reduced. The range of potentials within which this decrease took place coincided with the affected zone in the voltagrams in solution by varying O_2_ availability (Figure 5). This would support what the article by Correia, et al. sets out [6]. As the humidity conditions also remain practically constant between both states as temperature does, this diminished current density would be related to reduced O_2_ availability. The voltagrams and cathodic Tafel representations in Figure 9 present the same morphology and effect with the O_2_ availability reductions on the three studied concretes due to the figure showing the results obtained in one of the w/c = 0.6 samples as an example.

Moreover, the logarithmic representation of the voltagrams (Figure 9b) helps to distinguish the two lines observed in solution which, as shown by a former study, have been associated with a reduction in the O_2_, H_2_O_2_, and H_2_O systems.

In the study in solution, line 1 is related to dissolved O_2_ availability, whereas line 2 is practically horizontal during the H_2_O reduction process. In the voltagrams presented in Figure 9, line 2 presents no horizontal trend section because we do not contemplate a current saturation case caused by the transport of H_2_O molecules towards the electrode.

**Figure 10:**
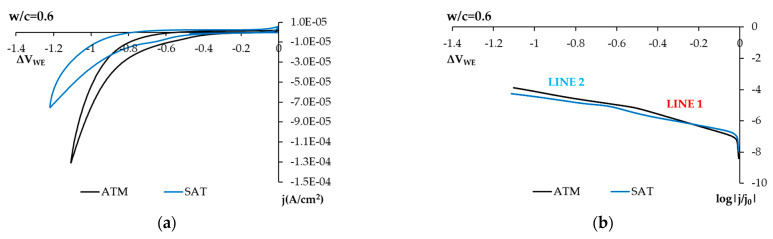

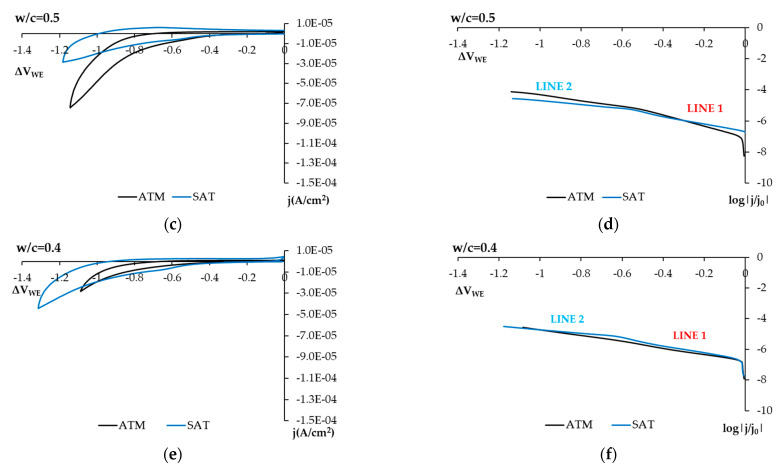
The cyclic voltammetry results obtained with the SS sensor in hardened concrete under atmospheric (ATM) and H_2_O-saturation (SAT) conditions using the 2-electrode technique. Ohm drop is corrected. (**a**) Voltagram of concrete w/c = 0.6. (**b**) Voltagram of cathodic sweeping represented logarithmically and corresponding to Figure (**a**), concrete w/c = 0.6. (**c**) Voltagram of concrete w/c = 0.5. (**d**) Voltagram of cathodic sweeping represented logarithmically and corresponding to Figure (**c**), concrete w/c = 0.5. (**e**) Voltagram of concrete w/c = 0.4. (**f**) Voltagram of cathodic sweeping represented logarithmically and corresponding to Figure (**e**), concrete w/c = 0.4.

Figure 10a,c,e shows the voltagrams obtained in both the saturated and non-saturated state ATM of the concrete pore system for one concrete sample w/c = 0.6, one concrete sample w/c = 0.5, and another sample w/c = 0.4. As shown, when the capillary system is under H_2_O saturation conditions, the peaks associated with the reduction in Fe(OH)_2_ and FeOOH layers (around −0.7 V) can be seen, especially in less porous concrete. Under the H_2_O-saturated concrete sample conditions, the conditions of the sensor’s environment are similar to the study in solution conditions, which is why the same peaks are noted.

The logarithmic representation (Figure 10b,d,f) of samples in the H_2_O saturation state shows that line 2 is not horizontal, but its slope tends to diminish. The same can be stated of line 1. This indicates less O_2_ availability according to that studied in solution.

**Figure 11:**
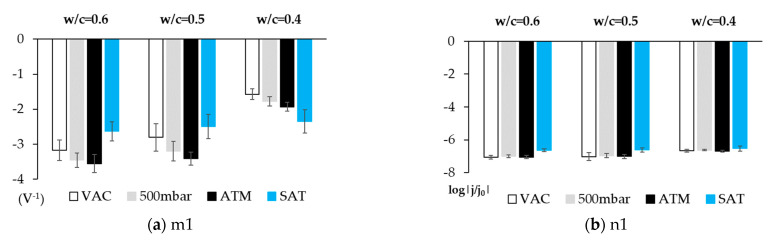
Average value of the slope (m1) and intercept (n1) of lines 1 and 2 for each studied dosage and each state. (**a**) Data of the slope of line 1 (m1). (**b**) Data of the intercept of line 1 (n1).

In order to assess the sensor’s effectiveness in distinguishing the different O_2_ availability conditions by simple data processing techniques, the average data obtained from the slopes and intercepts of the lines associated with O_2_ availability and humidity per dosage and for each studied condition were compared (Figure 11 and Figure 13).

Figure 11 depicts the value of the slope (m1) and the intercept (n1) for each studied dosage. The m1 values show a clear correlation between O_2_ availability and the slope obtained with the cathodic Tafel line. When examining the standard deviations, the results are not conclusive due to overlapping, but when we contemplate the minimum and maximum of the clearance range marked by them, we find that the correlation between O_2_ availability and the slope remains.

The data obtained for the non-saturated H_2_O samples (VACUUM, 500 mbar, and ATM) reveal that the parameter m1 value rises as O_2_ availability increases for all concretes. The highest absolute parameter m1 value is obtained in all concretes for higher O_2_ availability (ATM conditions), while the lowest absolute value is obtained under the VAC conditions (VAC, [O_2_] ≈ 0). This is consistent with that observed in solution. We also note that, from the results obtained in water-accessible porosity and mercury porosity (Table 4), the absolute parameter value for all the O_2_ levels in the less porous concrete (w/c = 0.4) is lower than that obtained in more porous concretes (w/c = 0.6 and w/c = 0.5) (Figure 11a).

**Figure 12:**
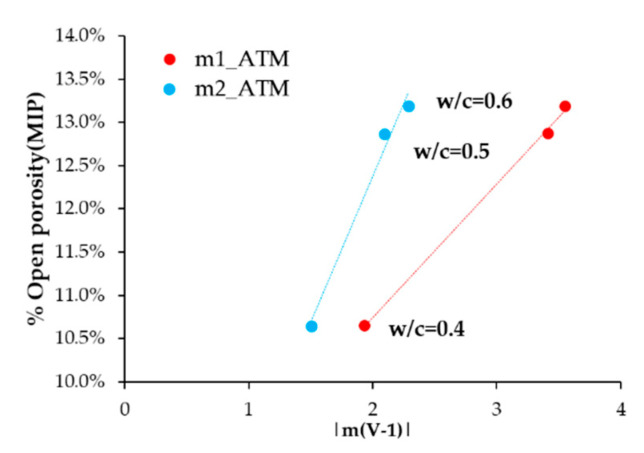
Correlation between the open porosity percentage (MIP) and slopes m1 and m2.

When studying the values of the slopes of lines m1 and m2 with the open porosity results according to MIP (Figure 12), a direct correlation appears. The greater slopes correspond to higher air porosities. This makes sense in light of the parameter m1 analysis, in which we identify a higher absolute value for this parameter with greater O_2_ availability.

For the saturated state, we observe that the value obtained for slope m1 (Figure 11a) is similar for all concretes (m1 = 2.53 ± 0.14 V^−1^), which might be due to the O_2_ concentration in H_2_O having a certain equilibrium value determined by the temperature of the system which, having lowered, must be transported from the outside by a winding network of saturated solvent capillaries.

Figure 11a depicts how for saturated concrete, the slope is practically the same for the three concretes. This agrees with the O_2_ concentration being the same regardless of the porosity-independent one (because gas solubility in H_2_O only depends on temperature). Although porosity affects the H_2_O/metal contact surface, parameter m1 does not depend on the electrode’s surface (Appendix A).

The results of the intercept of line 1 (n1), which are provided in Figure 11b, have similar values for all the studied cases, which allows us to consider that this value could be related to the exchange kinetics parameters of the metal/O_2_ system corrosion process.

**Figure 13:**
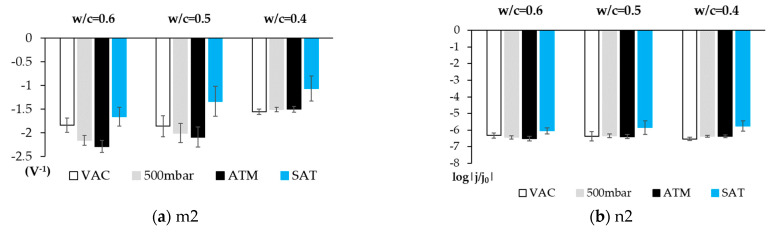
Average value of the slope (m2) and the intercept (n2) of line 2 per studied dosage and for each state. (**a**) Data on the slope of line 2 (m2). (**b**) Data on the intercept of line 2 (n2).

The interpretation of the results obtained from the line 2 analysis is complex. Figure 13a shows a similar behavior to that noted for line 1. The slope of the second section depends on O_2_ availability, while the intercept can practically be considered to take a constant value. Nevertheless, the tests in solution show that line 2 is practically horizontal and does not vary with variations in O_2_ availability. A proposal to justify the horizontal section observed when O_2_ in solution was reduced was previously mentioned. As H_2_O is now no longer the majority agent of the capillary system, we cannot expect it to impose its kinetic behavior on the system. The argument to justify the observed facts means having to revise the reaction mechanisms set out in the bibliography.

**Figure 14:**
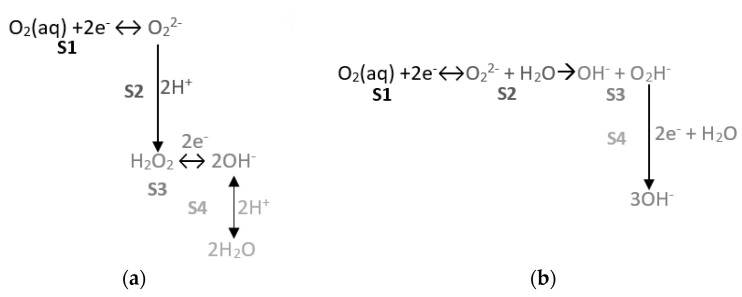
(**a**) Reaction of O_2_ reduction in acid/neutral medium. (**b**) Reaction of O_2_ reduction in basic medium. S: Stages of the O_2_ reduction reaction.

It is accepted that the O_2_ reduction reaction occurs by means of a sequence of different stages. For neutral or acidic media, the most frequently proposed process is that described in Figure 14a. As we are dealing with a basic medium (the pore solution pH of a recent concrete is higher than or equals 12.5), the reaction we contemplate is that illustrated in Figure 14b, instead of H_2_O_2_ appearing and, bearing in mind that proton availability would be low, the perhydroxyl species appearing. We propose that the slope of line 2 seen in the Tafel representation depends on the second reaction process, during which the perhydroxyl radical reacts with two electrons and one H_2_O molecule to give three hydroxide anions.

In accordance with the outline shown in Figure 14b, the slope of the second section depends on O_2_ availability, as noted from the data in Table 5. When the porous concrete system is saturated, O_2_ availability is low because of scarce O_2_ solubility in H_2_O and the slow speed of transporting O_2_ from the outside to the surface of the metal embedded in the material. Thus, the second process occurs to a lesser extent than when the porous system contains only residual humidity.

Therefore, the slope of line m2 diminishes, which also occurs with the slope of line 1, but without the horizontality that takes place when testing in solution. This last point is logical because, in the potentials region, H_2_O and O_2_ reduction processes overlap and pore saturation does not suffice to impose H_2_O reduction kinetics on HO_2_^−^ species reduction kinetics.

The slope m2 value is higher with increasing porosity (Figure 13a). The higher porosity is, the more favorable diffusion phenomena are. This fact allows the kinetics of reactions, reagents, and products to move more easily. Therefore, the stages 2, 3, and 4 of the O_2_ reduction reaction are more favored the higher the porosity is.

The interpretation of the intercept of line 2 (n2) (Figure 13b) is more difficult because it must correspond to a commitment dynamics state situation between the speed with which the HO_2_^−^ species is generated and the dismutation process that competes with the intermediate reduction to be transformed into OH^−^. As with n1, its value is similar for all states, but is slightly lower when concrete samples are H_2_O-saturated.

## 4. Discussion

Although it is true that the standard deviation of the m1 and m2 parameters among the various O_2_ availabilities is high, the little discrimination power can be solved by data analysis techniques following work protocols (RNA, PCA, and PLS). We can establish more accurate models to quantify O_2_ and humidity availability inside a hardened concrete matrix. Moreover, with these methods, we can include as many study variables as we wish. Furthermore, if we consider that the sensor responds to the presence of chlorides and carbonates, we could study their feasibility for also detecting the presence of Cl^−^ and concrete carbonation, which are the main precursor agents of steel rebar depassivation.

Furthermore, voltammetric SS sensors will allow us to define more economic sensor networks than one of noble metals. They can be made with a bigger surface which would provide a more robust response. We could install more control points which would allow more information to be collected because the cost of the material would lower, allowing us to intensify the statistical control which is a key factor to achieving success in strategies such as the SHM (Structural Health Monitoring). Moreover, being able to present resistance to different aggressive environments and respond to changes in the concrete matrix which compromise structures’ durability (variation in O_2_ availability, presence of chlorides, and concrete carbonation) offer a very high potential for monitoring structures.

According to this, the voltammetric SS sensors could be suitable to develop sensor systems to evaluate the initiation and propagation period of the rebars corrosion processes in the reinforcement of concrete structures.

## 5. Conclusions

Firstly, in accordance with the obtained results, parameters m1 and m2, the slopes of the lines identified logarithmically in the voltagram for the cathodic sweeping obtained with the SS sensor, are related to the different O_2_ reduction stages on the sensor’s surface. This allows us to assess the availability of both O_2_ and humidity in hardened concrete matrices.

Secondly, the parameters m1 and m2 present a direct correlation with the results of the standardized porosity tests and, with them, these concrete characterization parameters can be assessed in situ in RCS.

Thirdly, the voltametric behavior observed in dissolution agreed with the proposed O_2_ mechanism in Figure 14a.

Fourthly, the behavior of the sensor embedded in concrete is similar to the behavior in alkaline dissolution, but considering the differences due to the diffusion phenomena, limitations or transport limitations occurred in hardened concrete matrix.

Finally, as was demonstrated in the dissolution study, the SS sensor is sensitive to the presence of chlorides and carbonates.

## Figures and Tables

**Table 1:** Composition of the employed concretes.

Materials	kg/m^3^_concrete_		
	w/c = 0.6	w/c = 0.5	w/c = 0.4
Cement I 42.5 R-SR5 Water Superplastificiser	315	385	490
	189	193	196
	2.2	2.7	34
Silica sand Coarse aggregate	1212	1179	1115
	653	635	601

w/c: water/cement ratio.

**Table 2:** Table summarizing the number of samples per dosage.

w/c	Number of Masses	Number of Samples/Mass	Total No. Samples
0.6	3	2	6
0.5	3	2	6
0.4	3	2	6
		TOTAL	18

**Table 3:** Study phases. Schedule.

NON-SATURATED STATE	SEQUENCE 1: samples dosage w/c = 0.6	Samples were tested per dosage under the ATM, 500 mbar, and VAC conditions. Each sample was tested 3 times in each state.
	SEQUENCE 2: samples dosage w/c = 0.5	
	SEQUENCE 3: samples dosage w/c = 0.4	
SATURATED STATE	SEQUENCE 4: samples dosage w/c = 0.6, w/c = 0.5 and w/c = 0.4	All the samples were tested under SAT conditions. Each sample was tested 3 times in this state

**Table 4:** Standardized tests results. (**a**) Mean compressive strength values at 28 days (f_ck28days_) (UNE 12390-3:2009), %H_2_O accessible porosity (W.A.P) and %absorbed H_2_O (Abs.Water) (UNE 83980) and H_2_O penetration depth under pressure (W.P.D) (UNE 83-309-90). (**b**)air permeability coefficient (k) (UNE 83981), electrical resistivity (ρ) (UNE 83988-1:2008), and %Total pores MIP (ASTM-D4404-10).

	**f_cK28days_ (MPa)**	**CV**	**%W.A.P**	**CV**		**%Abs. H_2_O**	**CV**	**W.P.D (mm)**	**CV**
w/c = 0.6	34.5	6.03%	19.19%	13.79%		7.59%	6.81%	47	17.71%
w/c = 0.5	42.9	5.93%	17.21%	6.04%		7.47%	7.32%	26	3.10%
w/c = 0.4	56.8	4.50%	14.85%	2.59%		6.65%	4.68%	10	17.63%
(a)									
	**f_cK28days_ (MPa)**	**k (×10^−18^ m^2^)**			**CV**	**ρ (Ωm)**	**CV**	**%Total Pores MIP**	
w/c = 0.6	34.5	667.76			18.82%	50.68	12.88%	13.2%	
w/c = 0.5	42.9	521.37			3.45%	52.38	11.37%	12.9%	
w/c = 0.4	56.8	413.56			12.64%	62.56	9.88%	10.6%	
(b)									

f_ck28days_: Mean compressive strength values at 28 days; %W.A.P: H_2_O accessible porosity, %Abs H_2_O: %absorbed H_2_O, W.P.D: Water Penetration Depth under pressure. f_ck28days_: Mean compressive strength values at 28 days; k: air permeability coefficient, ρ: electrical resistivity, %Total pores MIP: Porosity obtained with Mercury injection porosity (MIP) test.

**Table 5:** Values of lines 1 and 2 defined in Figure 5b,c for the airless and aired atmospheres.

	Line 1			Line 2		
	Line Range	m1	n1	Line Range	m2	n2
Airless atmosphere (Ref.SCE)	−0.34 V to −0.870 V	−2.630 V^−1^	−6.920	−0.940 V to −1.220 V	−0.006 V^−1^	−4.468
Airless atmosphere (Ref.OCP)	−0.110 V to −0.61 V	−2.630 V^−1^	−6.336	−0.770 V to −0.880 V	−0.006 V^−1^	−4.467
Aired atmosphere (Ref. SCE)	−0.400 V to −0.800 V	−4.272 V^−1^	−7.388	−1.210 V to −0.880 V	−0.010 V^−1^	−3.879
Aired atmosphere (Ref.OCP)	−0.100 V to −0.460 V	−4.272 V^−1^	−6.052	−0.630 V to −0.920 V	−0.010 V^−1^	−3.876

**Table 6:** This table summarizes data for the lines related to the O_2_ availability and H_2_O results shown in Figure 6b.

pH	LINE 1			LINE 2		
	Range	m1 (V^−1^)	n1 (log|A/cm^2^|)	Range	m2 (V^−1^)	n2 (log|A/cm^2^|)
13	−0.46 V a −0.79 V	−6.0452	−8.6277	−0.82 V a −1.1 V	−0.2934	−3.9496
12.72	−0.38 V a −0.78 V	−5.4329	−8.07202	−0.82 V a −1 V	−0.5868	−4.1606
11.35	−0.43 V a −0.68 V	−7.0652	−8.5235	−0.74 V a −1.12 V	0.1537	−3.5363
8.35	−0.24 V a −0.40 V	−7.3822	−7.1177	−0.46 V a −0.9 V	−0.1207	−3.992
σ	-	0.9	0.69	-	0.31	0.26

σ = standard deviation.

**Table 7:** This table summarizes data for the lines related to Figure 8b.

	Whithout H_2_O_2_		0.02 m H_2_O_2_	
	m	n	m	n
LINE 1	−5.388	−8.041	−7.037	−8.183
LINE 2	−0.608	−4.180	0.0149	−3.412
LINE 3			−3.438	−6.471
LINE 4			−2.112	−5.012

## Appendix A

Considering that j = i/S:

(A1) $$m=\frac{\log\left(\frac{\frac{i_{2}}{S}}{\frac{i_{1}}{S}}\right)}{\Delta E_{WE-Ref1_{2}}-\Delta E_{WE-Ref1_{1}}}=\frac{\log\left(\frac{i_{2}}{i_{1}}\right)}{\Delta E_{WE-Ref1_{2}}-\Delta E_{WE-Ref1_{1}}}$$

In Equation (A1) we can see how the parameter does not depend on the electrode’s surface.

In order to see how this parameter does not depend on the reference, we considered expressing our results in accordance with a second reference (Ref2). To do so, we had to express the potentials referring to our Ref1 in relation to Ref 2, which was done by subtracting the difference of the existing potential measured between Ref1 and Ref2, by connecting Ref2 to the multimeter negative pole (or COM) from the potential expressed in Ref1.

(A2) $$m=\frac{\log\left(\frac{\frac{i_{2}}{S}}{\frac{i_{1}}{S}}\right)}{\Delta E_{WE-Ref2_{2}}-\Delta E_{WE-Ref2_{1}}}=\frac{\log\left(\frac{i_{2}}{i_{1}}\right)}{\left(\Delta E_{WE-Ref1_{2}}-\Delta E_{Ref1-Ref2}\right)-\left(\Delta E_{WE-Ref1_{1}}-\Delta E_{Ref1-Ref2}\right)}$$

By removing parentheses and simplifying the previous expression, we obtained:

(A3) $$m=\frac{\log\left(\frac{i_{2}}{i_{1}}\right)}{\Delta E_{WE-Ref1_{2}}-\Delta E_{WE-Ref1_{1}}}$$

As Equation (A3) remains the same as Equation (A1), it shows that the parameter did not depend on either the reference with which we expressed the potentials or the electrode’s surface. However, as the parameter depended directly on the intensities ratio, we were unable to compare the parameters obtained with the different electrode configurations because field intensities differed. In other words, we cannot compare the measurements taken with the 2-electrode configuration to those obtained with the 3-electrode ones.
